# The Chinese Herbal Medicine Formula Sheng-Fei-Yu-Chuan-Tang Suppresses Th2 Responses and Increases IFN**γ** in *Dermatophagoides pteronyssinus* Induced Chronic Asthmatic Mice

**DOI:** 10.1155/2013/984121

**Published:** 2013-03-14

**Authors:** Chia-Hung Lin, Ching-Hua Yeh, Li-Jen Lin, Jen-Shu Wang, Shulhn-Der Wang, Shung-Te Kao

**Affiliations:** ^1^Graduate Institute of Chinese Medicine, China Medical University, Taichung 40402, Taiwan; ^2^Graduate Insitute of Medical Science, College of Health Science, Chang Jung Christian University, Tainan 71101, Taiwan; ^3^School of Chinese Medicine, College of Chinese Medicine, China Medical University, Taichung 40402, Taiwan; ^4^Institute of Medical Science, Tzu Chi University, Hualien 97004, Taiwan; ^5^School of Post-Baccalaureate Chinese Medicine, College of Chinese Medicine, China Medical University, Taichung 40402, Taiwan; ^6^Department of Chinese Medicine, China Medical University Hospital, Taichung 40402, Taiwan; ^7^School of Chinese Medicine, College of Chinese Medicine, China Medical University, Taichung 40402, Taiwan

## Abstract

Sheng-Fei-Yu-Chuan-Tang (SFYCT), a traditional Chinese medicine formula consisting of 13 medicinal plants, has been used in the treatment of asthma. This study demonstrated the immunoregulatory effect of SFYCT on chronic allergic asthma using the *Dermatophagoides-pteronyssinus- * (Der p-) challenged chronic asthmatic murine model. SFYCT decreased the airway hyperresponseness (AHR), pulmonary inflammatory cell infiltration, and airway remodeling in Der p mice. SFYCT treatment decreased Der p-induced total IgE and Der-p-specific IgG1 but not IgG2a/2b Ab titer in serum of Der p mice. SFYCT also decreased Th2 cytokines, IL-4, IL-5, and IL-13, but increased IFN-**γ** and IL-12 in the BALF of Der p mice. TGF-**β**1 and collagen production in the lung of mice were decreased by SFYCT. The mRNA expression of chemokine including Eotaxin, RANTES, and MCP-1 in the lung of Der p mice was decreased by SFYCT. In conclusion, the suppressed Der-p-induced airway inflammation, remodeling, and hyperresponseness in chronic asthma murine model are related to SFYCT inhibits Th2 responses, decreases chemokine expression and promotes IFN-**γ** and IL-12 production. SFYCT could show Der-p-induced Th2 responses to Th1 responses by increasing IFN-**γ** which is merit for clinical application on asthma patients.

## 1. Introduction

Allergic asthma, an acute-on-chronic inflammatory disease, is a worldwide public health problem because of the rapidly increasing prevalence [1]. The characteristics of allergic asthma induced by inhaled allergens or nonspecific stimuli include airway eosinophilia, goblet cell hyperplasia with mucus hypersecretion, collagen deposition, and smooth muscle cell hypertrophy in lung, subepithelial thickening, and hyperresponsiveness in airway [2]. T-cell subsets, T helper 1 (Th1) and T helper 2 (Th2), response to allergens and regulate immune reactions during asthma. Asthma is considered a Th2-cell-driven inflammatory disease [3]; thus, drugs that can suppress Th2 cytokine production would prove useful as allergen immunotherapy agents [4]. However, antiasthmatic medicines, such as corticosteroids or *β*-agonists, help chronic asthmatic patients to inhibit asthmatic symptoms but not to cure the disease [5]. These agents cause serious side effect, overall immune suppression which results in increased susceptibility to infections, particularly in children [6, 7]. Thus, more and more asthmatic patients starting to use complementary and alternative medicine [8]. Traditional Chinese medicines (TCMs) have been used in treating asthma for centuries in Asia [9]. Some herbal formulas, herbal derivatives, and TCMs have provided scientific evidence supporting the use of treating allergic asthma by immune-regulatory effects [10–13]. These findings suggest a great potential in the development of herbal interventions to treat allergic asthma.

Sheng-Fei-Yu-Chuan-Tang (SFYCT), a formula on the basis of an empirical traditional Chinese medicine prescription composite of 13 medicinal plants (Table 1), has been used to treat bronchial asthma for decades in the Veterans General Hospital, Taichung, Taiwan. In the present study, the therapeutic effect on asthmatic syndrome of SFYCT was investigated in a *Dermatogoides-pteronyssinus-* (Der-p-) induced allergic asthma murine model [14]. Repeatedly exposing BALB/c mice to Der p via intratracheal (i.t.) exposure induces lymphocyte proliferation, Th2 cytokine release, airway inflammation, and remodeling [12]. Th2 cytokines, IL-4, IL-13, and IL-5 produced by activated CD4^+^ T cells, play a central role in the pathogenesis of allergic asthma [15]. IFN*γ* is a key cytokine in bridging the innate and the adaptive arms of the immune system and helps the development of a Th1-type response [16]. Because asthma is associated with dysregulated Th2 responses, enhanced Th1 responses may suppress the development of allergic airway inflammation. Therefore, strategies that enhance Th1 responses or increase IFN*γ* production have been proposed as therapies for ameliorating allergic airway inflammation [17, 18]. In the present study, we investigated the immunoregulatory effect of SFYCT on Der-p-induced chronic asthmatic murine model. Most treatments including corticosteroids and TCMs reduce AHR and airway inflammation by inhibiting Th1 responses (IFN-*γ*/IgG2a) as well as Th2 (IL-4, IL-5, and IL-13/IgE) responses [4, 8, 9, 11–13, 19]. However, SFYCT suppressed Th2 cytokines but elevated IFN-*γ* and IL-12 production. To our knowledge, this is the first TCM formula, SFYCT, documented supresses pulmonary allergic reactions through skewing Der p-induced Th2 responses to Th1 responses by increasing IFN-*γ* and IL-12. The present study demonstrates that SFYCT may offer some clinical advantages over corticosteroids because it is less likely to increase the patient's susceptibility to infection.

## 2. Materials and Methods

### 2.1. Mice and Reagents

Specific pathogen-free, male, 6 wk old BALB/c mice from the National Laboratory Animal Center, ROC, were housed in a microisolator cage and fed sterile food and water* ad libitum*. All experimental animal care and treatment followed the guidelines setup by the Institutional Animal Care and Use Committee of the China Medical University. Lyophilized house dust mites (*Dermatophagoides pteronyssinus* (Der p)) were purchased from Allergon (Engelholm, Sweden). Crude mite preparation was extracted with ether. After dialysis with deionized water, the mite extract was lyophilized and stored at −70°C until use. LPS concentration of the Der p preparations was 1.96 EU/mg of Der p (Limulus amebocyte lysate test; E-Toxate; Sigma-Aldrich).

### 2.2. SFYCT Preparation

SFYCT (batch number 98041021) was supplied by Koda Pharmaceuticas Ltd. (Taoyuan, Taiwan). The preparation was a mixture of 13 Chinese herbal medicines shown in Table 1. In brief, these were extracted with 1 L of boiled water twice for 1 hr. Poaching liquid was mixed two times. The dregs of the decoction were removed after filtering. The filtered liquid was lyophilized and crushed into a thin powder. The yield of the dried extract was about 38%. SFYCT was dissolved in distilled water and stored at −20°C before administration to mice.

### 2.3. Der p Challenge and Assessment of Airway Inflammation

 In Der-p-challenged BALA/c mice (*n* = 6), allergic airway inflammation and remodeling were provoked by subjecting mice to i.t. administered Der p (1 mg/mL, 50 *μ*L) in phosphate-buffered saline (PBS) once a week for 4 weeks (total 5 doses). In the SFYCT treated mice, mice were gavaged with SFYCT (1 g/kg) 30 min before Der p challenge. In parallel experiments, normal mice were gavaged with distilled water and i.t. administered PBS as control group. Mice were killed by i.p. injection of xylazine (200 *μ*g/mice) and ketamine (2 mg/mice) 3 days after the last challenge. Bronchoalveolar lavage fluid (BALF) was performed (two washes of 1 mL of ice-cold endotoxin-free PBS) according to a previously described procedure [11]. Serum and BALF were collected and stored at −80°C for further analysis. Differential counts were performed on cytospin preparations (1 × 10^5^ cells/100 *μ*L of BALF) stained with Liu's stain reagents (Biotech, Taiwan) in a blind manner after total leukocyte counting. 

### 2.4. Measurement of Airway Hyperresponsiveness

The methacholine-induced pause value was used in live mice as a marker of airway responsiveness (AHR) to bronchoconstrictors. AHR was measured in mice using a single-chamber, whole-body plethysmograph (Buxco Electronics, Inc., Troy, NY, USA) according to the manufacture's protocol. The enhanced pause (Penh) variable was used to estimate airway resistance. Mice were serially exposed to increasing doses of nebulized methacholine (0, 3.125, 6.25, 12.5, 25, and 50 mg/mL) (Sigma-Aldrich, St. Louis, MO, USA) in PBS for 3 minutes, respectively, and Penh values were measured for 3 minutes following the end nebulization of methacholine.

### 2.5. Histology Examination

Paraffin-embedded lung was cut into 5 *μ*m sections and stained with H&E or periodic-acid-Schiff (PAS) stain. Light microscopy was used for histologic assessment. The degree of inflammatory changes was evaluated with a semiquantitative scale of 0–5 for inflammatory cell infiltration, perivascular spaces, and peribronchial spaces. The scale was graded as follows: 0 (none), 1 (minimal, <1%), 2 (slight, 1–25%), 3 (moderate, 26–50%), 4 (moderate/severe, 51–75%), and 5 (severe/high, 76–100%) [20].

### 2.6. Collagen Analysis

The lung tissue (100 mg) of each mice was homogenized mechanically in 2 mL HBSS. The collagen contents of the lung homogenates were analyzed using Sircol collagen assay kit (Biocolor, Belfast, UK).

### 2.7. Flow Cytometric Analysis

Monoclonal antibodies including PE and/or FITC-conjugated anti-mouse CD4, FITC-conjugated anti-mouse CD8, PerCP-conjugated anti-mouse CD3, and FITC-conjugated anti-mouse CD25 (BD Pharmingen) were used for cell fluorescence staining. BALF cells (1 × 10^5^) were stained with mAb for 30 min on ice. After washing, stained cells were quantified by FACScan (Becton-Dickinson Immunocytometry system, San Jose, CA, USA).

### 2.8. Measurement of Der-p-Specific IgG1, IgG2a/2b, and IgE

An IgE-specific ELISA was used to measure the total IgE Ab levels in serum using matching mAb pairs (BD PharMingen) according to the manufacturer's instructions. A450 readings of the samples were converted to ng/mL using a standard curve generated with double dilutions of mouse IgE isotype standard (BD PharMingen). For Der-p-specific Abs, serum samples were added in duplicate onto ELISA plates coated with Der p (2 g/mL in 0.1 M NaHCO_3_, pH 8.3). After incubation overnight at 4°C, the plates were washed and incubated with biotinylated rat anti-mouse IgG1 or IgG2a/2b monoclonal Ab (2 g/mL; BD PharMingen) for 1 h, followed by washings with PBS and the addition of streptavidin-HRP conjugate (1 : 1000 dilution, BD PharMingen). The plates were washed and developed with a tetramethylbenzidine microwell peroxidase substrate system (Kirkegaard & Perry Laboratories, Gaithersburg, MD) and read at OD 450.

#### 2.8.1. Measurement of Cytokine Levels Concentration

Cytokine concentration was measured by a sandwich ELISA technique. Mouse IL-4, IL-5 ELISA Ready-SET-Go (eBioscience, San Diego, CA), IL-12, IL-13, IL-17, INF-*γ*, and TGF-*β* ELISA DuoSet (R&D System, Abingdon, UK) were used to detect cytokine concentrations according to the manufacturer's protocol. 

### 2.9. Reverse Transcription-Polymerase Chain Reaction

Reverse transcription-polymerase chain reaction (RT-PCR) was performed to determine the Eotaxin, RANTES, monocyte chemotactic protein (MCP)-1 or *β*-actin mRNA expression. Total RNA of lung was extracted using Trizol solution (Life Technologies) and subjected to reverse transcription with StrataScript H-reverse transcriptase (Strata-gene, La Jolla, CA, USA) to generate cDNA. Gene-specific primer pairs (sense and antisense, resp.) used are as follows: RANTES, F5′-AGAAGTGGGTTCAAGAATACAT-3′ and R5′-GGACCGAGTGGGAGTAG-3′; Eotaxin, F5′-ACATGTTACATTTAAGAAATTGGAGTT-3′ and R5′-AGGTCAGCCTGGTCTAC-3′; MCP-1, F5′-ACCTGCTGCTACTCATTCAC-3′ and R5′-TACAGAAGTGCTTGAGGTGG-3′; *β*-actin, F5′-GCTGGAAGGTGGACAGCGAG-3′ and R5′-TGGCATCGTGATGGACTCCG-3′. PCR products were electrophoresed on 1.5% agarose gels and stained with ethidium bromide. *β*-Actin amplification was used as an internal control. The relative quantity of PCR products is expressed as fold increase relative to *β*-actin.

### 2.10. Statistical Analysis

Data are presented as means ± SE. Differences between mean values were estimated using a Student's *t*-test. A *P* value < 0.05 was considered significant. For comparisons of data that were not normally distributed, a Mann-Whitney *U* test was performed.

## 3. Results

### 3.1. Effects of SFYCT on Airway Inflammation and Hyperresponsiveness in Der p Mice 

**Figure 1:**
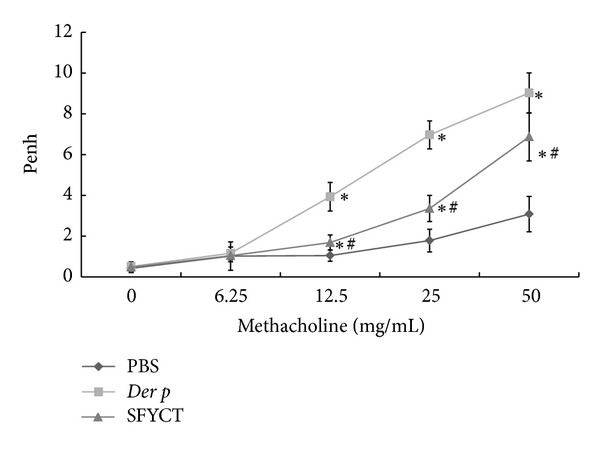
The suppressive effects of SFYCT on airway hyperresponsiveness in repetitive Der-p-challenged mice. Methacholine-induced airway hyperresponsiveness was determined at day 3 after the last challenge. Values represent the means ± SE of 6 mice. *P < 0.05 compares with Naive group; ^#^ *P* < 0.05 compares with Der p group.

Most allergic asthmatic patients are sensitized by house dust mite allergens, such as Der p [14]. Thus, repetitive Der p challenge protocol described in Section 2 was used to induce chronic airway inflammation in mice. Repeatedly exposing BALB/c mice to Der p via intratracheal (i.t.) exposure induces lymphocyte proliferation, Th2 cytokine release, airway inflammation, and remodeling [12]. In the present study, 3 days after the last challenge, the numbers and percentages of macrophages, neutrophils, eosinophils, and lymphocytes in the BALF of nontreated Der p mice significantly higher than those of control mice (Table 1). AHR, determined using Penh values, in Der p mice was higher than that in control mice (Figure 1). The results showed clear signs, inflammatory cells infiltration and AHR, of chromic asthmatic mice model. SFYCT decreased the absolute number of inflammatory cells but did not changed their percentages in the BALF of Der p mice (Table 1). SFYCT also decreased the Penh value in Der p mice (Figure 1). 

**Figure 2:**
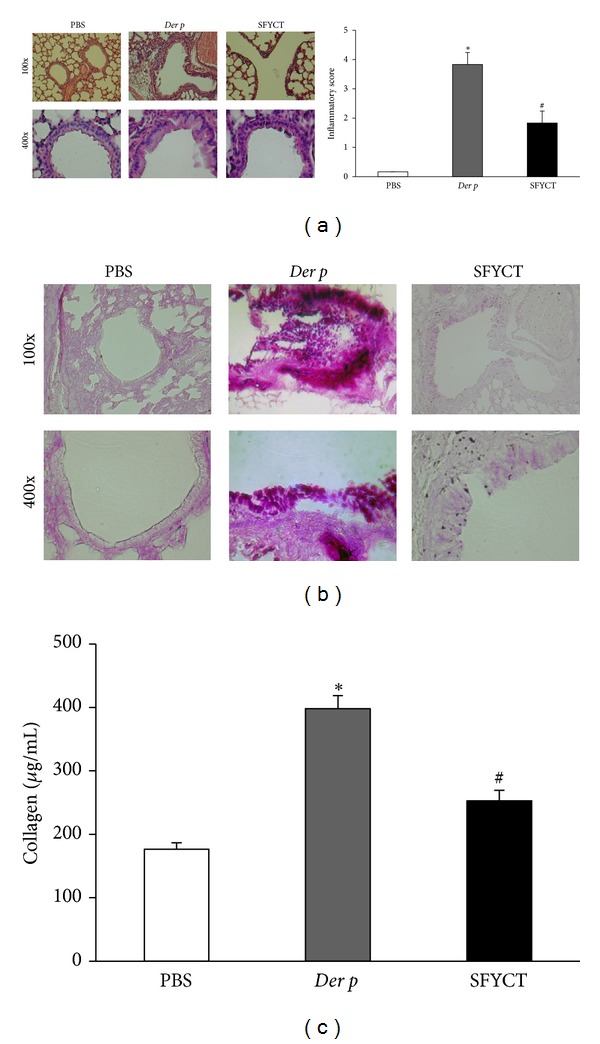
The effects of SFYCT on Der-p-induced airway inflammation, goblet cell hyperplasia, mucus hypersecretion, and collagen deposition in lung tissue of mice. (a) H&E stain and inflammatory score show the histopathologic change and inflamatory cell infiltration around the blood vessels of mice. (b) PAS stain shows the mucus of goblet cells in the airway of mice. Goblet cell hyperplasia and mucus plug in airway from PBS sham treated mouse and nontreated or SFYCT treated Der p mouse. (c) Collagen levels in the lung of mice were determined as described in Section 2. Values represent the means ± SE of 6 mice. *P < 0.05 compares with Naive group; ^#^ *P* < 0.05 compare with Der p group.

The inflammation degree and pathological change in the lung of mice were observed. There was no pulmonary inflammation in normal mice, but widespread peribronchiolar and perivascular infiltrates (Figure 2(a)) as well as matrix deposition in subepithelial regions accompanied with abrogation of mucus production by hyperplastic goblet cells (Figure 2(b)) were shown in the lung of Der p mice. The semiquantitation of inflammatory changes in the lung of Der-p mice is higher than that of normal mice (Figure 2(a)). The collagen content, represented the levels of matrix within the lung tissue, in Der p mice, was higher than that in control mice (Figure 2(c)). SFYCT treatment inhibited inflammatory cell infiltration, decreased matrix and mucus deposition, and collagen content in the lung of Der p mice (Figures 2(a)–2(c)). These results showed that SFYCT could attenuate AHR in Der p mice and protects against allergenic airway inflammation, goblet cell activation, and collagen deposition.

### 3.2. Effects of SFYCT on Inflammatory Cell Number and Cellular Distributions in BALF of Der p Mice

The total and various cell counts in BALF from normal mice and Der p mice with or without SFYCT treatment were analyzed (Table 2). In the BALF of normal mice, there was few macrophages, lymphocytes, or neutrophil but no eosinophil was detected. In Der p mice, all kinds of inflammatory cell numbers in BALF were markedly increased but SFYCT treatment significantly decreased them.

**Figure 3:**
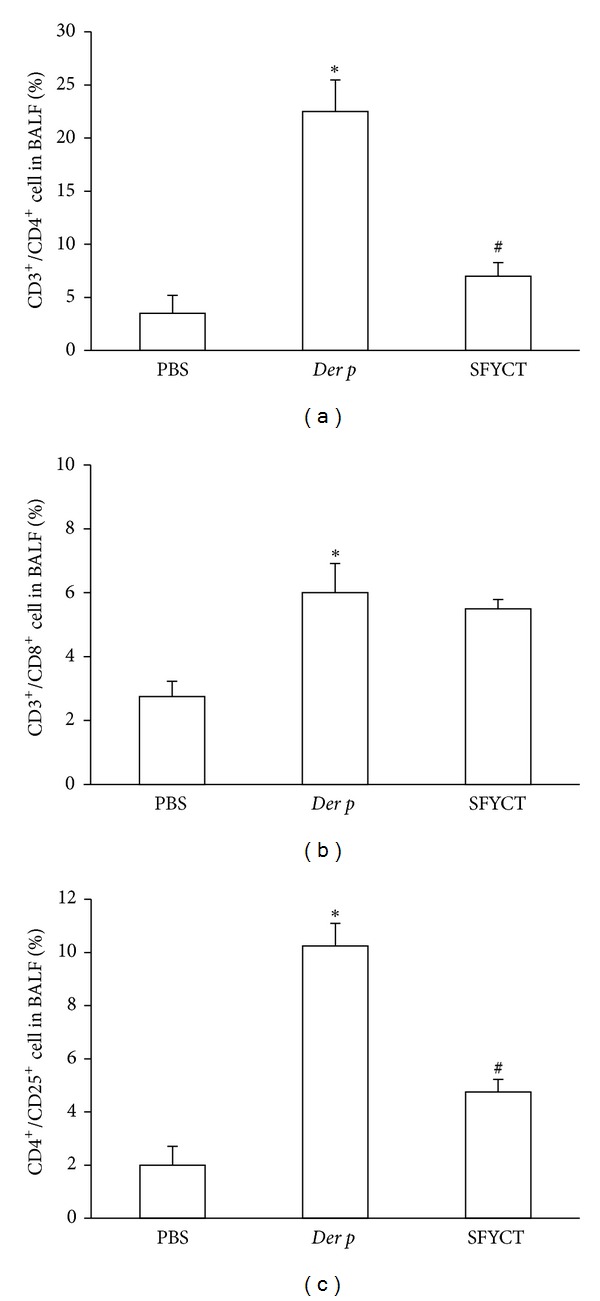
The effect of SFYCT on T-cell subsets in BALF of Der p mice. (a) CD3^+^/CD4^+^, (b) CD3^+^/CD8^+^, and (c) CD4^+^/CD25^+^ lymphocyte levels were determined by flow cytometry with immunofluorescence of monoclonal antibodies. Values represent the means ± SE of 6 mice. *P < 0.05 compares with Naïve group; ^#^ *P* < 0.05 compare with Der p group.

The T-cell subset distribution in the BALF of mice was determined by flow cytometry. The percentages of CD3^+^/CD4^+^, CD3^+^/CD8^+^, and CD4^+^/CD25^+^ lymphocytes in Der p mice were significantly higher than in normal mice. SFYCT treatment decreased the CD3^+^/CD4^+^ and CD4^+^/CD25^+^ lymphocyte percentage but barely affected CD3^+^/CD8^+^ lymphocyte in BALF of Der p mice (see Figure 3).

### 3.3. Effects of SFYCT on Cytokine in Serum and BALF of Der p Mice

**Figure 4:**
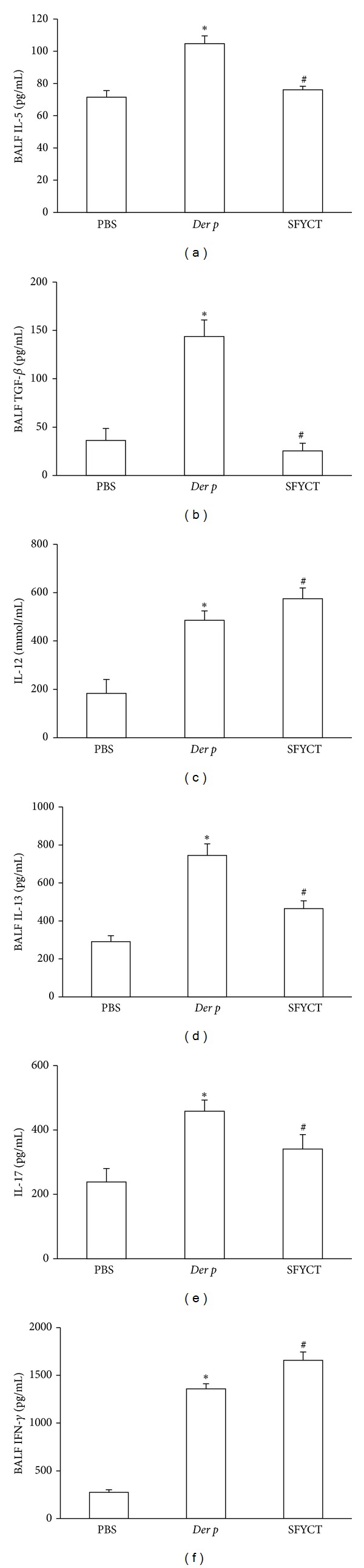
The effects of SFYCT on inflammatory mediators in the BALF of Der p mice. The levels of (a) IL-5, (b) TGF-*β*, (c) IL-12, (d) IL-13, (e) IL-17, and (f) IFN-*γ* were determined by ELISA. Values represent the means ± SE of 6 mice. *P < 0.05 compares with Naïve group; ^#^ *P* < 0.05 compares with Der p group.

**Figure 5:**
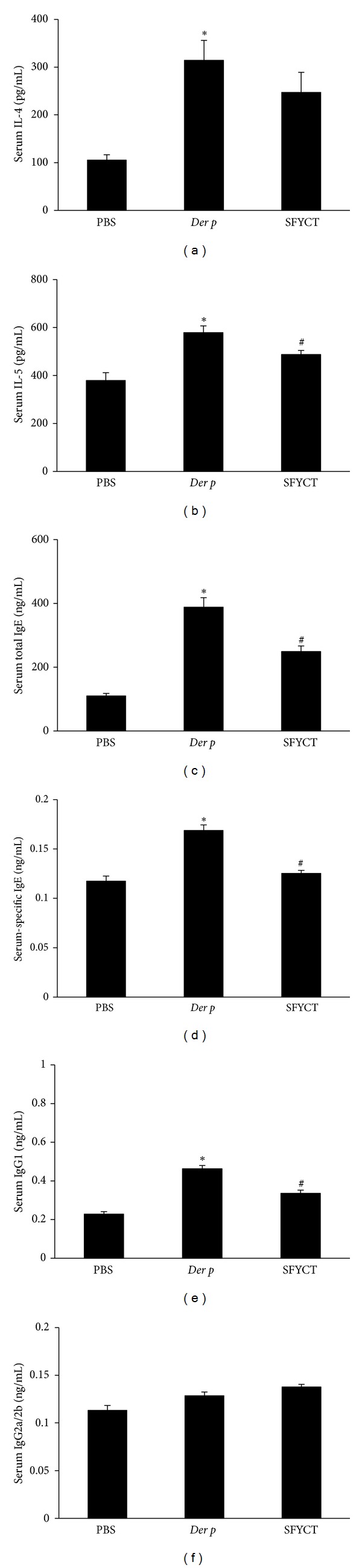
The evaluation of cytokine secretion and the antibody titers from the sera of mice. The effects of SFYCT on IL-4, IL-5 ((a), (b)), or allergen-specific Ab concentrations (c)–(f) were evaluated at day 3 after the last challenge in the serum of Der p mice. Values represent the means ± SE of 6 mice. *P < 0.05 compares with Naïve group; ^#^ *P* < 0.05 compares with Der p group.

To determine the possible effect of SFYCT on T-cell responses, the levels of T-cell cytokine concentration and Ab titers in the BALF or serum of Der p mice were analyzed by ELISA. SFYCT treatment significantly decreased the levels of Der p-induced cytokine, IL-5, IL-13, IL-17 and TGF-*β*, but enhanced IFN*γ* as well as IL-12 secretion in BALF of Der p mice (Figure 4). The elevated serum levels of IL-4 and IL-5 in Der p mice were reduced by SFYCT (Figure 5). Furthermore, the serum levels of total IgE and Der-p-specific IgE in mice were increased after repeated Der p challenge and SFYCT treatment reversed the phenomenon. The levels of IgG1 normally associated with a Th2 immune response while IgG2a/2b associated with a Th1 immune response [5, 21]. The serum titers of IgG1 and IgG2a/2b Abs were elevated in Der p mice suggesting a mixed Th1/Th2 response. SFYCT treatment decreased the IgG1 but not IgG2a/2b Ab titer in serum of Der p mice (Figure 5).

### 3.4. Effect of SFYCT on Chemokine Expression in the Lung of Der p Mice

**Figure 6:**
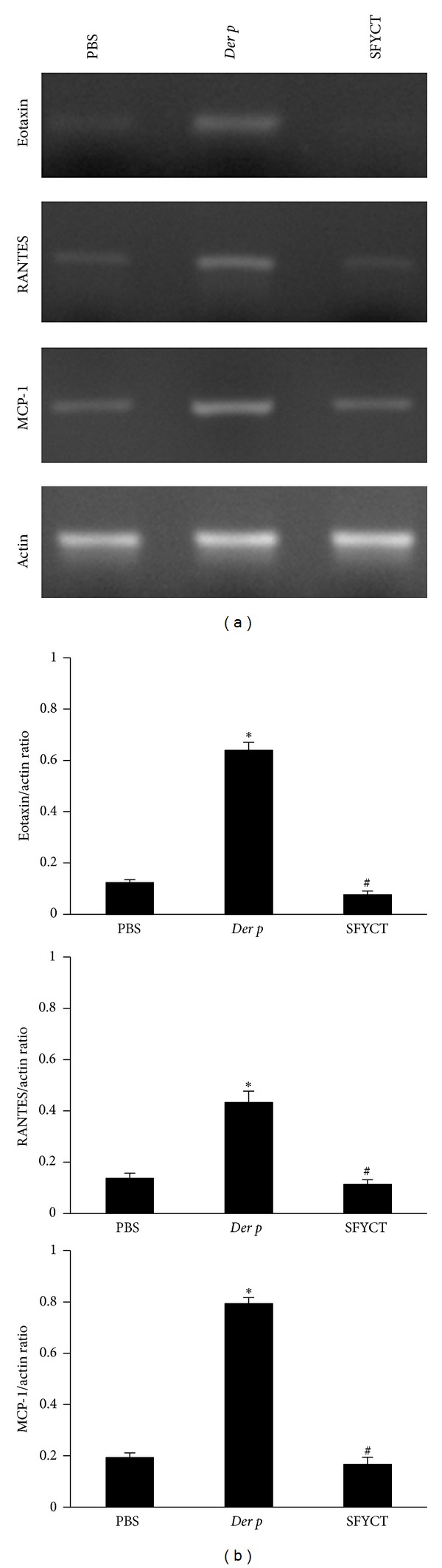
The effects of SFYCT on chemokine expression in the lung of mice. The mRNA expression of Eotaxin, RANTES, and MCP-1 in the lung of mice was evaluated by RT-PCR. *β*-Actin mRNA expression was included as internal control. (a) shows the representative experiment and (b) shows the quantification of mRNA levels expressed as mean ± SE (*n* = 6 per group). *P < 0.05 compares with Naïve group; ^#^ *P* < 0.05 compares with Der p group.

The mRNA expression of chemokines including Eotaxin, RANTES, and MCP-1 in the lung of mice was analyzed by RT-PCR. The mRNA expressions of these chemokines were higher in Der p mice than those in normal mice. SFYCT treatment significantly inhibits the increased Eotaxin, RANTES, and MCP-1 mRNA in the lung of Der p mice (see Figure 6).

## 4. Discussion

TCMs have been reported with therapeutic effects on allergic asthma [19, 22]. SFYCT, a formula designed following the traditional Chinese medicine theories and clinical experience, has been used to treat asthmatic patients in Taiwan for decades. Unlike the side effect from using corticosteroids, SFYCT relieves asthmatic syndrome without total immune suppression. In present study, the immunoregulatory effects and possible mechanism of SFYCT were investigated in Der p-induced chronic allergic asthma murine model. SFYCT treatment suppressed air way inflammation, AHR, and Th1 response but increased IFN*γ* and IL-12 production in asthmatic mice.

SFYCT exhibited nonspecific anti-inflammatory property with reducing the cell number of all kinds of inflammatory cells in the BALF of Der p mice. Pathological observations also showed that SFYCT reduced inflammatory cell infiltration. Airway remodeling, including lamina thickening and airway structural changes, a central feature of asthma, is closely related to progression of AHR [21, 23]. TGF-*β* not only regulates cellular biological processes leading to airway remodeling [24] but also contributes to increased collagen synthesis and AHR [25]. Treatment with the antibody to TGF-*β* reduced the number of mucus-secreting goblet cells in a murine model of asthma [26]. Although corticosteroids and *β*2 agonists are able to improve the management of asthma, they are ineffective at inhibiting TGF-*β* to reverse the structural remodeling of airways, especially in patients with chronic asthma [27–29]. SFYCT treatment decreased the TGF-*β* production in BALF and collagen synthesis in the lung of Der p mice. Taken together, these results suggest that treatment with SFYCT can suppress AHR by decreasing airway inflammation and mucus hypersecretion associated with TGF-*β* secretion. The properties of SFYCT with anti-inflammation, decreasing airway remodeling, and inhibiting AHR promise this formula an effective therapeutic modality for asthma.

Immunoglobulin E (IgE), an important mediator of allergic reactions, plays a central role in airway inflammation and asthma-related symptoms. Anti-IgE therapies have the potential to block an early step in the allergic cascade [21]. In the serum of Der p mice, high level of Der-p-specific IgG1 Ab, is associated with a Th2 immune response [30]. By contrast, increasing IgG2a production is considered to be beneficial for asthma treatment [31]. SFYCT treatment significantly decreased Der-p-specific IgE and IgG1 but slightly increased IgG2a/2b in the serum of Der p mice. These data suggested that the benefit of SFYCT treatment might be related to inhibiting Th2 response. Furthermore, Th2 cell play an important role in orchestrating the asthmatic inflammatory response [32]. The flow cytometry analysis showed that SFYCT treatment decreased the percentage of the CD3^+^/CD4^+^ T-cell subset in BALF but increased the CD3^+^/CD8^+^ T-cell subset. These data suggested that SFYCT could modulate the Th-cell differentiation from Th2-cell dominant to Th1-cell dominant in the airway of chronic asthmatic mice. 

IFN*γ* is a key cytokine in bridging the innate and the adaptive arms of the immune system and helps the development of a Th1-type response [16]. SFYCT increased the IFN-*γ* secretion in the BLAF of Der p mice. This immunoregulation may be more beneficial than Th1 cytokine (IFN-*γ* and IL-12) or Th1 adjuvant therapy, which may cause undesirable inflammation because of higher-than-normal levels of Th1 cytokines [33]. IL-12, produced by antigen-presenting cells, promotes differentiation of Th1 cells, IFN-*γ* production, and inhibits differentiation of Th0 cells into IL-4–secreting Th2 cells [34]. Because SFYCT induced IFN-*γ* and IL-12 as well as reduced IL-4 and IL-5 production in BALF, whether this effect was dependent on IL-12 should be further investigated.

Corticosteroids are the most powerful nonspecific anti-inflammatory drugs routinely used to treat asthma. However, it is also well known that corticosteroids produce overall immune suppression, which results in increased susceptibility to infections. Corticosteroid immunosuppression is due to induction of T-lymphocyte apoptosis [6]. In this study, SFYCT decreased the cell number of macrophage, neutrophil, and eosinophil but not lymphocyte in BALF of Der p mice. FASCs results also showed that SFYCT did not decrease the distribution of CD3^+^/CD8^+^ T-cell subset. These results suggest that SFYCT is not toxic to all lymphocytes, especially Th1-related lymphocyte. These findings, together with SFYCT suppressing Th2 cytokines accompanied with increasing IFN-*γ* secretion, clearly demonstrate that SFYCT actions on T cells differ from corticosteroids and suggest that SFYCT might be of more benefit to asthma patients.

Th2 cytokines play a central role in the pathogenesis of asthma. IL-4 or IL-13 promotes B-cell switching to IgE production and mucus hypersecretion. IL-5 has been shown to be the primary determinant of eosinophil priming, activation, recruitment, and survival [15, 16]. Anti-IL-4 or anti-IL-13 receptor antibodies could suppress antigen-induced AHR but not eosinophilic inflammation [35, 36]. AHR is regulated by integrated IL-13, IL-4, and IL-5 signals [37]. Compared with the sham treated group, SFYCT treatment decreased three major Th2 cytokines, IL-4, IL-5, and IL-13, production in serum or BALF in Der p mice. It seems that SFYCT is offering advantage over therapeutic administration of single antibodies against IL-4, IL-5, or IL-13 or their receptors since natural allergic airway reactions are mediated by a combination of Th2 cytokines. Furthermore, IL-17 was demonstrated as indispensable to induce granulocyte influx into the lung in allergic asthma model [38, 39]. IFN-*γ* is indicated to limit the IL-17-producing T-cell population [40]. IL-17 is mainly produced by macrophages in allergic inflammation related to asthma [41]. We found that IL-17 production and macrophage infiltration were attenuated while IFN-*γ* production was increased in SFYCT treated mice. These observations suggest that SFYCT could limit the IL-17 related immune response by increasing IFN-*γ* production.

After asthma attack, Eotaxin and RANTES are chemoattractants for eosinophils [42] while RANTES and MCP-1 are involved in recruiting monocytes [43, 44] from system to lung. Asthma-relevant chemokines, mentioned above, have been targeted by humanized blocking mAb to their receptors or removal of chemokines via soluble receptors or small molecule receptor antagonists [44]. Here, SFYCT decreased the mRNA expression of Eotaxin, RANTES, and MCP-1 in the lung of Der p mice which may contribute to the reduction in eosinophils and monocyte recruitment in airway.

In conclusion, SFYCT suppressed Der-p-induced airway inflammation, remodeling, and hyperresponseness in chronic asthma murine model. The effect was accompanied by inhibiting Th2 responses and decreasing chemokine expression but elevating IFN-*γ* and IL-12 production. This is the first study of TCM formula, SFYCT, documented that may attenuate asthma symptoms through skewing Der-p-induced Th2 responses to Th1 responses by increasing IFN-*γ* and IL-12. SFYCT provides more clinical advantages over corticosteroids for asthma treatments.

## Figures and Tables

**Table 1:** The ratio of the components in SFYCT.

Components	Amount (g)
Ginseng Radix (root of *Panax ginseng* C. A. Meyer)	4
Atractylodis Ovatae Rhizoma (root and rhizome of *Atractylodes macrocephala Koide*)	4
Citri Reticulatae Pericarpium (skin of fruit of *Citrus reticulate Blanco*)	4
Ephedrae Herba (stem of *Ephedrae sinica* STAPF)	1.2
Mori Ramulus (branch of *Morus alba L.*)	4
Radix Bupleuri (root of *Bupleurum chinense DC*)	4
Cinnamomi Ramulus (root of *Cinnamomum cassia* BL)	4
Scutellariae Radix (root of *Scutellaria bicalensis* George)	4
Schizonepetae Herba (stem of *Schizonepeta tenuifolia* Briq)	6
Sileris Radix (root of *Siler divaricatum* Benth et Hook f.)	6
Glycyrrhizae Radix (root of *Glycyrrhiza uralensis* Fisch)	4
Zingiberis Recens Rhizoma (root and rhizome of *Zingiber officinale* Rosc.)	2
Zizyphi Sativae Fructus (fruit of *Zizyphus jujube* Mill. Var. inermis Rehd.)	6

Total amounts	53.2

**Table 2:** The total cell number and cellular distributions in BALF of mice 72 h after repetitive Der p challenge.

	Total cells (×10^4^/mL)	Macrophages (%)	Lymphocytes (%)	Neutrophils (%)	Eosinophils (%)
PBS	16.5 ± 1.73	15.57 ± 1.85	0.61 ± 0.22	0.32 ± 0.22	0
		(94.36 ± 11.20)	(3.68 ± 1.33)	(1.95 ± 1.36)	(0)
Der p	71.25 ± 4.43*	50.39 ± 3.35*	3.81 ± 1.69*	13.16 ± 1.63*	4.28 ± 0.27*
		(70.72 ± 4.70)	(5.34 ± 2.37)	(18.46 ± 2.29)	(6.00 ± 0.37)
SFYCT	40.25 ± 3.5^∗#^	27.19 ± 1.53^∗#^	2.97 ± 1.67	8.87 ± 2.22^∗#^	1.41 ± 0.27^∗#^
		(67.54 ± 3.81)	(7.38 ± 4.15)	(22.02 ± 5.50)	(3.50 ± 0.66)

Values represent the mean ± SE of 6 mice. *P < 0.05 compares with naïve; ^#^ *P* < 0.05 compares with Der p.
